# Epstein–Barr virus and multiple sclerosis: the dawn of a new age

**DOI:** 10.1002/cti2.1457

**Published:** 2023-06-27

**Authors:** Tri Giang Phan

**Affiliations:** ^1^ Precision Immunology Program Garvan Institute of Medical Research Sydney NSW Australia; ^2^ St Vincent's Healthcare Clinical Campus, Faculty of Medicine and Health, School of Clinical Medicine UNSW Sydney Sydney NSW Australia

## Abstract

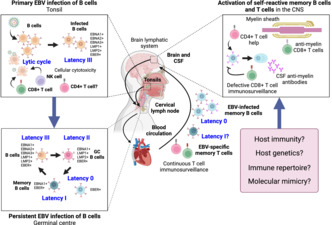

Multiple sclerosis (MS) is a chronic, disabling disease of the central nervous system (CNS) that affects 2.8 million people worldwide (35.9 per 100 000 population).^1^ The symptoms and signs of disease result from plaques of CNS demyelination, presumably from autoimmune attack of the myelin sheath. In 1868, in a series of brilliant lectures, Jean‐Martin Charcot at the Salpêtrière Hospice in Paris pieced together the clinical, anatomical and pathophysiological features of a new disease entity associated with *sclérose en plaques* in the brain and spinal cord, a condition, which would later be translated into English as multiple sclerosis.^2^ Since this Victorian era description, a number of observations have supported the notion that MS may arise from an aberrant immune response to an unidentified infectious agent. These include the presence in > 90% of people with MS (PwMS) of oligoclonal bands of immunoglobulin G (IgG) in the cerebrospinal fluid (CSF), which are often also found in CNS infections, for example with bacterial, cryptococcal and tuberculous meningitis, viral encephalitis and neurosyphilis.^3^ However, oligoclonal bands in the CSF are also observed in patients with inflammation from systemic lupus erythematosus (SLE), neurosarcoidosis and traumatic brain injury.

Several candidate infectious agents have been proposed over the years as the trigger for MS. These include human herpes family viruses such as human herpes virus 6 (HHV‐6) and Epstein–Barr virus (EBV), coronaviruses and human endogenous retroviruses (HERVs). The leading candidate has been EBV, and this is supported by many studies providing epidemiological, immunological and virological evidence for a role for EBV in MS (summarised by Soldan and Lieberman^4^). This evidence had been largely circumstantial until 2022 when a landmark paper established convincingly that EBV infection was necessary to cause MS. In an epic effort that exemplifies the power of mega cohorts in the age of big data, Bjornevik *et al*. longitudinally tracked 10 million US military personnel over 20 years to show that the risk of MS increased 32‐fold after infection with EBV.^5^ The strength of this study lies not just in its scale, but also in the carefully designed questions and controls, which allowed the authors to draw such a powerful conclusion. However, EBV is ubiquitous and > 90% of the adult population worldwide is seropositive. The disconnect between the widespread prevalence of EBV and the infrequency of MS suggests that, while EBV may be necessary (Tier 1 of the Pender hypothesis^6^), it is not sufficient to cause MS and there are multiple additional genetic and environmental factors involved in disease pathogenesis (Figure 1).

**Figure 1:**
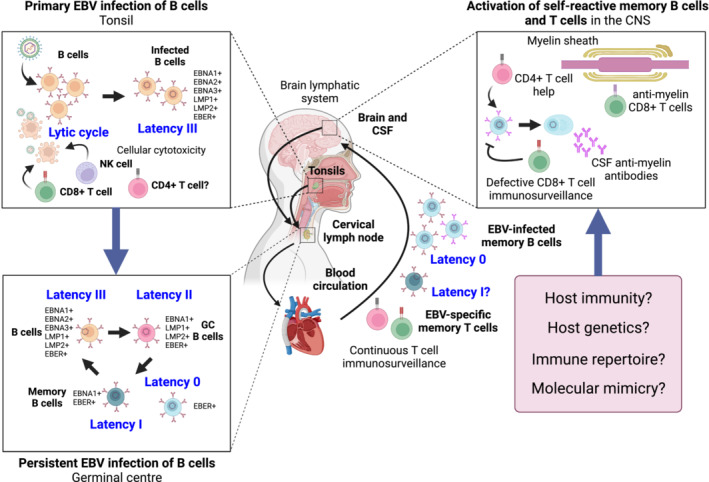
The EBV life cycle and MS. Primary EBV lytic infection of epithelial cells in the oral cavity is followed by latent infection of naïve B cells in the tonsils. In immunocompetent individuals, viral replication is controlled by NK and T cells. Infected naïve B cells migrate to the germinal centre where they are reprogrammed to become memory B cells. Latently infected memory B cells circulate via the blood and enter the CNS. Continuous immunosurveillance by EBV‐specific memory T cells suppresses viral replication. Through as yet undefined mechanisms, EBV‐infected memory B cells may activate anti‐myelin B cells and T cells to cause autoimmune attack of the myelin sheath. These mechanisms may include defective host immunity, for example, defective T‐cell immunosurveillance, host genetic susceptibility, immune repertoire (linked to the major histocompatibility complex class II antigen HLA‐DRB1*15:01), and molecular mimicry. This figure was created with BioRender.com.

In this Special Feature of *Clinical & Translational Immunology*, we have followed Charcot's interdisciplinary example and pieced together a collection of reviews by virologists, neurologists, immunologists and genomicists to get some perspective on the pressing unanswered questions arising from the resurgence of the ‘EBV hypothesis’ and what it means for PwMS. The Special Feature opens with Thomas, Rickinson and Palendira^7^ arguing, through the prism of EBV, that we need to move from the question of association, which appears settled, to questions of potential mechanisms, which remains largely unexplored. They review the EBV life cycle and how it intersects with host immune responses in both the lytic and latent phases of infection in B cells. This is particularly relevant as latent EBV infection of memory B cells may explain the efficacy of B‐cell depletion therapy in MS. Potential mechanisms discussed include defective T‐cell responses to EBV‐infected B cells, EBV transformation and rescue of autoreactive B cells from apoptosis and molecular mimicry between EBV‐derived antigens and CNS self‐antigens such as the cilial calcium‐activated chloride channel anoctamin‐2 (ANO2),^8^ glial cell adhesion molecule (GlialCAM)^9^ and α‐crystallin B (CRYAB).^10^ Thomas, Rickinson and Palendira identify several disconnects and missing links, which will hopefully be resolved by future mechanistic studies.

Next in the Special Feature, Afrasiabi *et al*. continue the search for disease mechanisms by interrogating the intersection between EBV and MS genetic risk loci.^11^ They describe ethnic differences in genetic susceptibility to MS and review data from genome wide association studies (GWAS) from the International Multiple Sclerosis Genetics Consortium, which has identified 32 major histocompatibility complex (MHC) and > 200 non‐MHC loci, which are estimated to contribute anywhere from 18.3% to 48% of the genetic risk. Cell‐specific gene regulatory network analysis revealed B cells and microglia as the key cells involved. They describe studies of EBV‐transformed lymphoblastoid cell lines (LCLs) that revealed interactions between EBNA2 and MS risk alleles that may control the EBV life cycle in latently infected B cells. Afrasiabi *et al*. speculate on the role of small interfering RNAs as a potential therapeutic option in early EBV infection to prevent the development of CNS autoimmunity. It will be interesting to see what additional insights will be provided by new genomic technologies, such as single cell eQTL mapping,^12^ and the analysis of cells from additional tissues beyond the blood such as deep cervical lymph nodes.^13^

This brings us to the third review in the Special Feature by Dyer *et al*., which spotlights the lessons from the clinical experience of treating PwMS.^14^ They describe the mechanisms of action, efficacy and impact on EBV of immunosuppressive disease‐modifying therapies such as B‐cell depletion with the anti‐CD20 monoclonal antibodies (rituximab, ocrelizumab and ofatumumab), drugs that block lymphocyte trafficking (natalizumab, fingolimod, siponimod and ozanimod), and ‘immune reconstitution therapy’ with cladribine, alemtuzumab and autologous hemopoietic stem cell transplantation (AHSCT). It is notable that the efficacy of some therapies does not appear to correlate with the levels of oligoclonal bands in the CSF, EBV‐specific antibodies in the serum or EBV‐specific T cells in the blood. Just as informative as the drugs that work are the drugs that exacerbate disease, such as lenercept, a recombinant fusion protein that blocks the TNF receptor, and atacicept, a recombinant fusion which blocks both B‐cell‐activating factor (BAFF) and a proliferation‐inducing ligand (APRIL). The latter depletes mature B cells, which require BAFF, and plasma cells, which require APRIL, but not memory B cells, which are able to survive independently of BAFF and APRIL. Dyer concludes by speculating on how new ideas and approaches in the postgenomics era might exploit these clinical insights to yield the next generation of therapeutics.

In the fourth review in the Special Feature, Lanz *et al*.^15^ provide a roadmap to show the way forward to understanding disease mechanisms and translating them to clinical trials for PwMS. They zero in on a region of the EBNA1 protein between amino acids 386 and 405 that includes a cross‐reactive sequence shared with GlialCAM,^9^ between amino acids 411 and 440 shared with ANO2 and between amino acids 411 and 426 shared with myelin basic protein. This hotspot for molecular mimicry in EBNA1 may have arisen by epitope spreading with three proteins that are in close physical proximity in the paranodal region of the axon. Lanz *et al*. argue that there is a need for antiviral trials and the development of vaccines, adoptive EBV‐specific T‐cell therapy and therapeutic RNAs against EBV to treat and prevent MS. In addition, they describe therapeutic RNA and DNA vaccines to tolerise the immune system against CNS self‐antigens in preclinical models and clinical trials in PwMS.

To round out the Special Feature, Smith and Khanna take up the Steinman challenge to describe the development of adoptive EBV‐specific T‐cell therapy for MS.^16^ This is particularly poignant as it comes in the footsteps of Michael Pender's championing of the EBV hypothesis for decades, long before it was fashionable. They describe repurposing of a therapy to treat MS that was initially designed to treat EBV‐associated malignancies in immunosuppressed patients. The technology depends largely on a deep understanding of the EBV life cycle to determine the optimal EBV antigens to target (EBNA1, LMP1 and LMP2). Smith and Khanna describe the long‐term clinical responses of the first few individuals treated with adoptive T‐cell therapy and the transition from bespoke autologous EBV‐specific T cell to a universal allogeneic ‘off‐the‐shelf’ product. This circumvented problems with failure to generate EBV‐specific T cells in some PwMS (Tier 3 of the Pender hypothesis) and also allowed manufacturing to be scaled up and commercialised. This product is now in a Phase 2 double‐blind, randomised, placebo‐controlled trial.

The papers by the Ascherio^5^ and Steinman laboratories^9^ in 2022 have invigorated MS research and opened up exciting new possibilities for MS research. More importantly, it has generated considerable hope and excitement in the MS community that we are watching the dawn of a new age. However, there is much work still to be carried out and many clinical questions to address. For example, while immune therapies have shown efficacy in many individuals with relapse‐remitting MS, not all respond. Furthermore, the progressive forms of MS remain stubbornly resistant to immune modulation. We hope that you will enjoy reading the articles in this Special Feature as much as we have enjoyed preparing them.

## Author Contributions

TGP drafted, edited and approved the manuscript.

## Conflict of Interest

The authors declare no conflict of interest.
